# Survival and reoperation in acute aortic syndromes—a single-centre experience of 912 patients

**DOI:** 10.1093/ejcts/ezad350

**Published:** 2023-10-25

**Authors:** Giacomo Murana, Gregorio Gliozzi, Paola Rucci, Daniela Votano, Valentina Orioli, Simona Rosa, Gianluca Folesani, Francesco Buia, Luigi Lovato, Davide Pacini

**Affiliations:** Division of Cardiac Surgery, Cardiac Surgery Department, IRCCS, Azienda Ospedaliero-Universitaria di Bologna, Bologna, Italy; Division of Cardiac Surgery, Cardiac Surgery Department, IRCCS, Azienda Ospedaliero-Universitaria di Bologna, Bologna, Italy; Department of Biomedical and Neuromotor Sciences, University of Bologna, Bologna, Italy; Division of Cardiac Surgery, Cardiac Surgery Department, IRCCS, Azienda Ospedaliero-Universitaria di Bologna, Bologna, Italy; Division of Cardiac Surgery, Cardiac Surgery Department, IRCCS, Azienda Ospedaliero-Universitaria di Bologna, Bologna, Italy; Department of Biomedical and Neuromotor Sciences, University of Bologna, Bologna, Italy; Division of Cardiac Surgery, Cardiac Surgery Department, IRCCS, Azienda Ospedaliero-Universitaria di Bologna, Bologna, Italy; Cardiovascular Radiology Unit, Cardio-Thoraco-Vascular Department, IRCCS, Azienda Ospedaliero-Universitaria di Bologna, Bologna, Italy; Cardiovascular Radiology Unit, Cardio-Thoraco-Vascular Department, IRCCS, Azienda Ospedaliero-Universitaria di Bologna, Bologna, Italy; Division of Cardiac Surgery, Cardiac Surgery Department, IRCCS, Azienda Ospedaliero-Universitaria di Bologna, Bologna, Italy; Department of Experimental, Diagnostic and Specialty Medicine, University of Bologna, Bologna, Italy

**Keywords:** Aortic, Acute aortic syndrome, Dissection, Penetrating ulcer, Intramural haematoma

## Abstract

**OBJECTIVES:**

Acute aortic syndromes are associated with poor outcomes, despite diagnostic and therapeutic advances. We analysed trends in volumes and outcomes from 2000 to 2021.

**METHODS:**

The study population includes 494 type A acute aortic syndromes (TAAAS) (54.2%) and 418 type B acute aortic syndromes (TBAAS) (45.8%). Primary outcomes were in-hospital mortality, long-term survival and freedom from aortic reoperation.

**RESULTS:**

Regardless the type of acute aortic syndrome, patient volumes increased over time. Patients with TBAAS were older, more likely to have comorbid conditions and previous cardiac surgery (*P* < 0.001), while cerebrovascular accidents were more frequent in TAAAS (*P* < 0.05). Among TAAAS, 143 (28.9%) required total arch and 351 (71.1%) hemiarch replacement. TBAAS management was medical therapy in 182 (43.5%), endovascular in 198 (47.4%) and surgical in 38 (9.1%) cases. Overall in-hospital mortality was 14.6% [18.2% in TAAAS (95% confidence interval (CI) 14.4–21.2%) vs 10.7% in TBAAS (95% CI 7.8%–13.7%); *P* = 0.0027]. After propensity score adjustment, in-hospital mortality exhibited a significantly decreasing trend from 2000 to 2021 (*P* < 0.001) in TAAAS and TBAAS. 1-, 5- and 10-year survival was 74.2%, 62.2% and 45.5% in TAAAS and 75.4%, 60.7% and 41.0% in TBAAS (*P* = 0.975), with no differences among treatment strategies. The adjusted cumulative reoperation risk at 10 years was more than two-fold in TBAAS versus TAAAS (9.5% vs 20.5%, hazard ratio (HR) = 2.30, 95% I 1.31–4.04).

**CONCLUSIONS:**

In the last decades, better patient triage and surgical/endovascular techniques led to substantial improvements in the management of acute aortic syndrome, with reduction in early mortality and reoperation rate. However, long-term mortality is still >50%.

**Figure:**
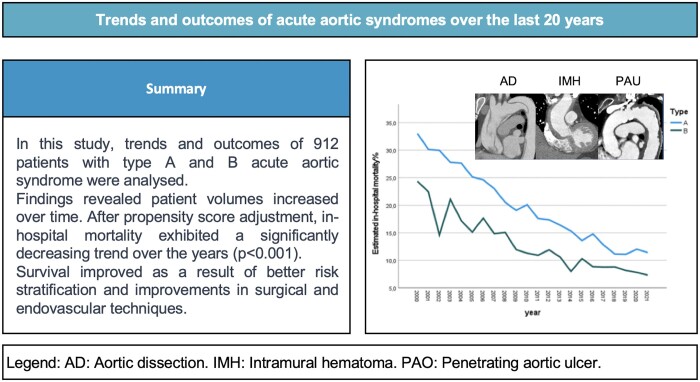

## INTRODUCTION

Acute aortic syndrome is an urgent life-threatening condition typically characterized by acute chest and/or back pain, which can be associated with 1 or more signs and symptoms of malperfusion. It includes classic aortic dissection, intramural haematoma and penetrating atherosclerotic aortic ulcer. An involvement of the ascending aorta is described in two-thirds of cases [type A acute aortic syndrome (TAAAS)], while the descending aorta alone is involved in one-third [type B acute aortic syndrome (TBAAS)]. Acute aortic syndrome is more frequent in males (66.9%) and is strong associated with hypertension (76.6%) [1]. When the ascending aorta is involved, the natural evolution of the disease is catastrophic, with an estimated mortality of 18 to 49%, increasing up to 2% per hour after symptoms onset [2]. Surgical treatment is undoubtedly the mainstay of treatment for TAAAS that reduces the early mortality rate to 10–25% [1, 3], although the extent of the repair both proximally and distally is still debated. The natural history of TBAAS depends on the presence of complications, such as malperfusion, rupture, recurrent or refractory pain or rapid aortic expansion, with mortality rates of about 16%, compared to 2.5% if uncomplicated [4].

The treatment of acute aortic syndrome has been one of the major challenges in cardiovascular surgery over the last decades, not only for the high mortality rates but also for the large involvement of different organs, necessitating high diagnostic and therapeutic skills. Surgical techniques for TAAAS have been improving for both proximal and distal repair, allowing more complete repair, especially in high volume centres. Optimal medical treatment is preferred for uncomplicated TBAAS, while complicated cases are treated either with endovascular or surgical treatment [5].

The aim of the present study is to investigate the trends of outcomes of TAAAS and TBAAS over 22 years in a single centre.

## MATERIALS AND METHODS

### Ethics statement

Patients were identified through comprehensive quality registries at IRCCS Azienda Ospedaliero-Universitaria di Bologna. These data were approved for the use in human subject by the institutional review board (IRB No. 121/2022/Disp/AUOBo) that waived the need for written informed consent because of anonymity.

### Patient population and study design

Our Institution is a tertiary referral centre for acute aortic syndrome in the metropolitan area of Bologna that includes 1 million inhabitants. Patients with acute aortic syndrome are carefully triaged by the on-call cardiac surgeon for centralization in Cardiac Intensive Care Unit and for further decision-making: total body computed tomography (CT) angiogram for anatomical assessment and surgical planning is mandatory for all patients, including unstable ones.

**Figure 1:**
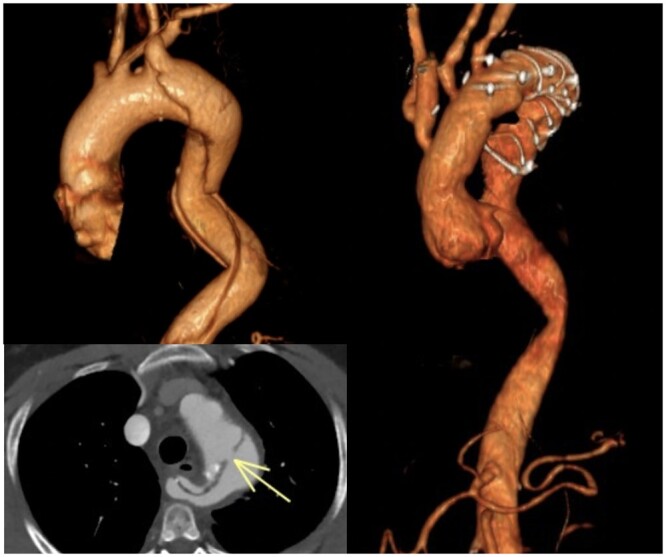
Computed tomography angiogram of a patient with type B acute aortic dissection treated with a Frozen Elephant Trunk.

The gold standard treatment for TAAAS is urgent/emergency surgery: each patient undergoes an individualized comprehensive evaluation by the cardiac surgeon: no exclusion criteria, such as old age or neurological signs/symptoms, are applied *a priori*. The extent of repair depends mainly on the location of primary entry tear, also considering the experience of the first surgeon and trying to predict the fate of the downstream aorta (Fig. 1). The primary objective of the repair is to resect the primary entry tear. As per the proximal repair, aortic root replacement was indicated in case of: involvement of >1 sinus of Valsalva, dilated aortic root, connective tissue disorders and coronary involvement. Regarding the distal repair, hemiarch replacement was the primary approach but, if the most proximal entry tear was located in the aortic arch, arch replacement was performed, with distal anastomosis either in zone 1, 2 or 3 and separate reimplantation of the epiaortic vessels. In addition, a frozen elephant trunk was usually preferred in case of distal malperfusion.

TBAAS definitive management is the result of a multidisciplinary and stepwise approach that involves Cardiac and Vascular Surgeons and Interventional Radiologists: endovascular or surgical treatment is performed only in complicated cases (with clinical malperfusion, rupture, recurrent or refractory pain or rapid aortic expansion) or in selected uncomplicated cases with high-risk features (aortic diameters >40 mm, radiological evidence of malperfusion, haemothorax), according to current guidelines [6]. The collaboration of the Aortic Team allows a highly tailored surgical approach for TBAAS.

Our institutional acute aortic syndrome registry was retrospectively reviewed from January 2000 to December 2021. Patients with TAAAS with a contraindication for surgery due to excessive surgical risk and those who died soon after admission were excluded from the analysis.

In-hospital mortality, 10-year survival and freedom from aortic reoperation were the primary outcomes.

After discharge, patients were routinely followed up by CT scan and outpatient clinic evaluation at 1, 6 and 12 months and then yearly, for both TAAAS and TBAAS. Patients not followed up at our institution, were contacted by phone.

### Statistical analysis

Categorical variables were summarized as absolute and percentage frequencies and continuous variables as mean ± standard deviation or median and interquartile range as appropriate. Shapiro–Wilk’s test was used to test the normality of the distribution of continuous variables. Categorical variables were compared between groups using chi-square or Fisher’s exact test and continuous variables using *t*-test (or Mann–Whitney test for non-normal variables), respectively. Time to event (death and reintervention) was estimated using Kaplan–Meier curves and was compared between groups using log-rank test. Data for survival analyses were censored at death or at the 10-year follow-up, whichever came first.

Due to the observational nature of the data, baseline patient characteristics differ between TAAAS and TBAAS. Propensity scores estimate the probability of being TAAAS over TBAAS based on observed baseline characteristics and can therefore, at least partially, account for the imbalance between the 2 groups.

The propensity score was estimated using logistic regression, with dissection type as regressed on the characteristics differing significantly between the 2 types. Multiple logistic regression was used to estimate in-hospital mortality by dissection type and year of recruitment, with propensity score adjustment.

The cumulative risk of reoperation in TAAAS versus TBAAS was estimated using competing risk analysis, with death as competing event and adjustment for the propensity score. The significance level was set at *P* < 0.05. IBM SPSS statistics 27.0 (Statistical Package for Social Science, IBM, Armonk, NY, USA) and Stata 15.1 (StataCorp LLC, College Station, Texas, USA) were used for statistical analyses.

## RESULTS

**Figure 2:**
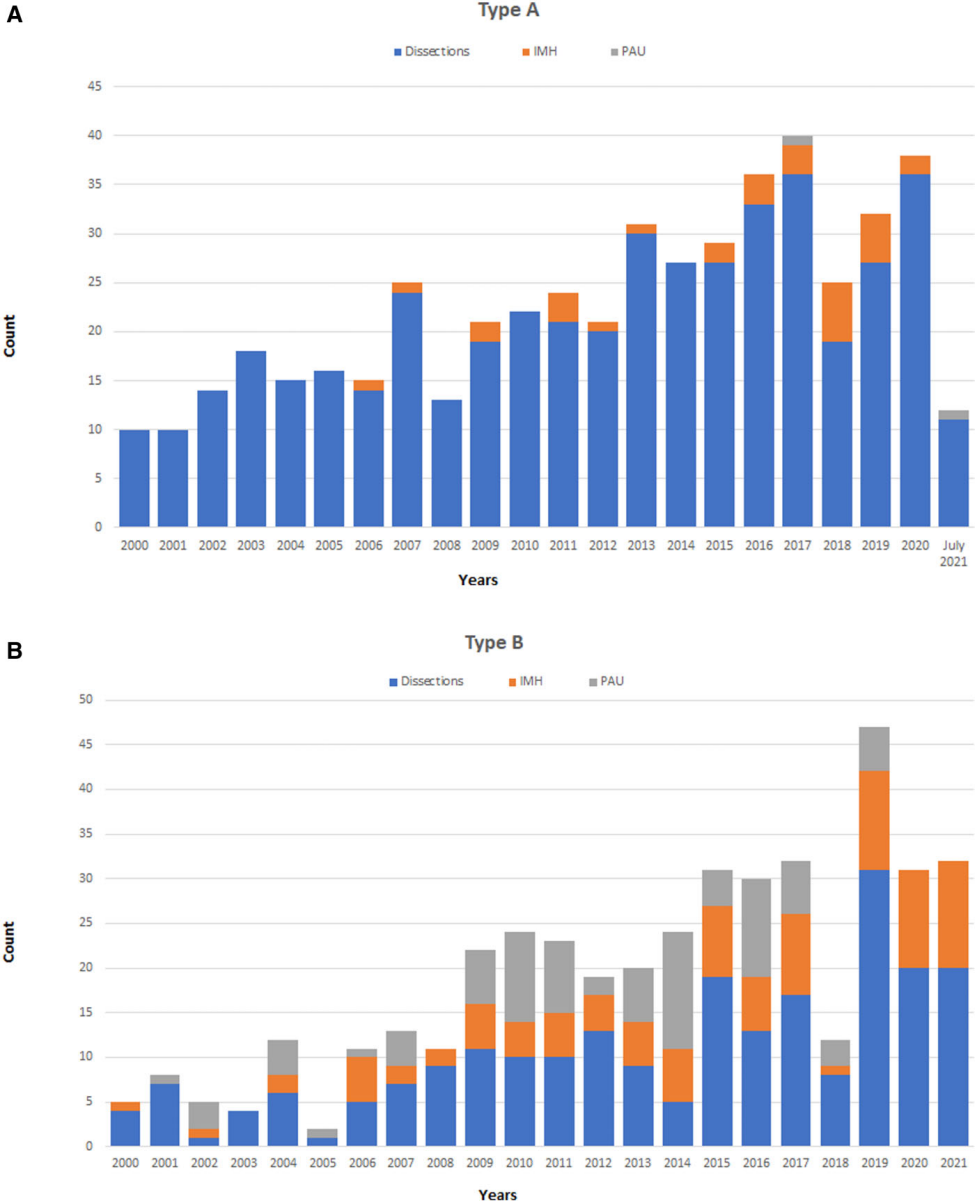
Acute aortic syndromes: type A acute aortic syndromes (**A**) and type B acute aortic syndromes (**B**) activity volumes per year from January 2000 to December 2021.

The study population includes 912 patients admitted with a diagnosis of AAS (symptoms onset <14 days), 494 (54.0%) affected by TAAAS, recruited between January 2000 and July 2021 and 418 (46.0%) by TBAAS, recruited between January 2000 and December 2021. Regardless the type of acute aortic syndrome, the number of referred patients increased over time, as shown in Fig. 2. The characteristics of patients with TAAAS and TBAAS were very similar between 2000–2010 and 2011–2021 (Table 1 and Supplementary Material, Table S1). However, in TAAAS, MI, AKI and coma were significantly less frequent in the second period as a result of improved referral and triage procedures.

**Table 1:** Patient characteristics in type A acute aortic syndromes and type B acute aortic syndromes between years 2000–2010 and 2011–2021

Baseline features	TAAAS			TBAAS		
	2000–2010 (*n* = 179)	2011–2021 (*n* = 315)	*P*-Value	2000–2010 (*n* = 117)	2011–2021 (*n* = 301)	*P*-Value
Age (years), mean (SD)	64.0 ± 11.9	66.2 ± 11.6	0.050	67.6 ± 13.2	68.7 ± 12.5	0.422
Age by type^a^						
AD, mean (SD)	63.9 ± 12.0	65.6 ± 11.8		62.9 ± 14.4	65.1 ± 13.3	
IMH, mean (SD)	68.9 ± 6.2	71.6 ± 7.7		74.6 ± 9.3	73.1 ± 10.5	
PAU, mean (SD)		74.3 ± 4.0		72.6 ± 8.3	72.9 ± 8.9	
BMI (kg/m^2^), mean (SD)	26.8 ± 4.5	26.5 ± 4.3	0.485	26.0 ± 3.3	26.5 ± 4.7	0.219
LVEF, mean (SD)	56.0 ± 6.7	59.3 ± 5.7	<0.001	59.7 ± 8.3	60.6 ± 6.5	0.244
Gender, male, *n* (%)	118 (65.9)	213 (67.6)	0.700	98 (83.8)	202 (67.1)	0.001
Hypertension, *n* (%)	139 (77.7)	232 (73.7)	0.323	93 (79.5)	252 (83.7)	0.306
Smoking history, *n* (%)	35 (19.6)	109 (34.6)	<0.001	44 (37.6)	135 (44.9)	0.179
Diabetes mellitus, *n* (%)	4 (2.2)	18 (5.7)	0.110	8 (6.8)	29 (9.6)	0.366
CAD, *n* (%)	14 (7.8)	19 (6.0)	0.444	17 (14.5)	27 (9.0)	0.096
TIA/stroke, *n* (%)	31 (17.3)	61 (19.4)	0.574	1 (0.9)	2 (0.7)	1,000
Previous cardiac surgery, *n* (%)	5 (2.8)	9 (2.9)	0.967	8 (6.8)	29 (9.6)	0.366
CKD, *n* (%)	11 (6.1)	21 (6.7)	0.821	23 (19.7)	34 (11.3)	0.025
COPD, *n* (%)	15 (8.4)	28 (8.9)	0.847	8 (6.8)	32 (10.6)	0.237
AR ≥ moderate, *n* (%)	49 (27.4)	99 (31.4)	0.344	3 (2.6)	14 (4.7)	0.418
Dyslipidaemia, *n* (%)	8 (4.5)	69 (21.9)	<0.001	30 (25.6)	107 (35.5)	0.503
Marfan syndrome, *n* (%)	3 (1.7)	1 (0.3)	0.138	3 (2.6)	6 (2.0)	0.715
Syncope at presentation, *n* (%)	17 (9.5)	30 (9.5)	0.992	6 (5.1)	19 (6.3)	0.647
Coma at presentation, *n* (%)	1 (0.6)	10 (3.2)	0.064	0 (0.0)	0 (0.0)	
Malperfusion, *n* (%)	50 (27.9)	73 (23.2)	0.240	44 (37.6)	85 (28.2)	0.063
Follow-up, *n* (%)						
PND	25 (14.0)	42 (13.3)	0.843	0 (0.0)	1 (0.3)	1,000
MI	8 (4.5)	4 (1.3)	0.034	3 (2.6)	4 (1.3)	0.405
AKI	70 (39.1)	76 (24.1)	<0.001	5 (4.3)	19 (6.3)	0.421
Dyalisis	51 (28.5)	73 (23.2)	0.190	5 (4.3)	11 (3.7)	0.767
SCI	1 (0.6)	2 (0.6)	1,000	3 (2.6)	5 (1.7)	0.691
Coma	17 (9.5)	15 (4.8)	0.040	0 (0.0)	0 (0.0)	

a Age in type A syndromes: ANOVA *F* = 4.77, *P* = 0.009, significant post hoc comparisons, IMH versus AD, *P* = 0.012; age in type B syndromes: ANOVA *F*: 28.5, *P* < 0.001; significant post hoc comparisons: IMH and PAU versus AD, *P* < 0.001.

AD: aortic dissection; AKI: acute kidney injury; ANOVA: analysis of variance; AR: aortic regurgitation; BMI: body mass index; CAD: coronary artery disease; CKD: chronic kidney disease; COPD: chronic obstructive pulmonary disease; IMH: intramural haematoma; LVEF: left ventricle ejection fraction; MI: myocardial infarction; PAU: penetrating aortic ulcer; PND: permanent neurological deficit; SCI: spinal cord ischaemia; SD: standard deviation; TAAAS: type A acute aortic syndromes; TBAAS: type B acute aortic syndromes; TIA: transient ischaemic attack.

Overall in-hospital mortality for acute aortic syndrome was 14.8% and it was higher for TAAAS (18.2% vs 10.7% in TBAAS; *P* = 0.001).

**Figure 3:**
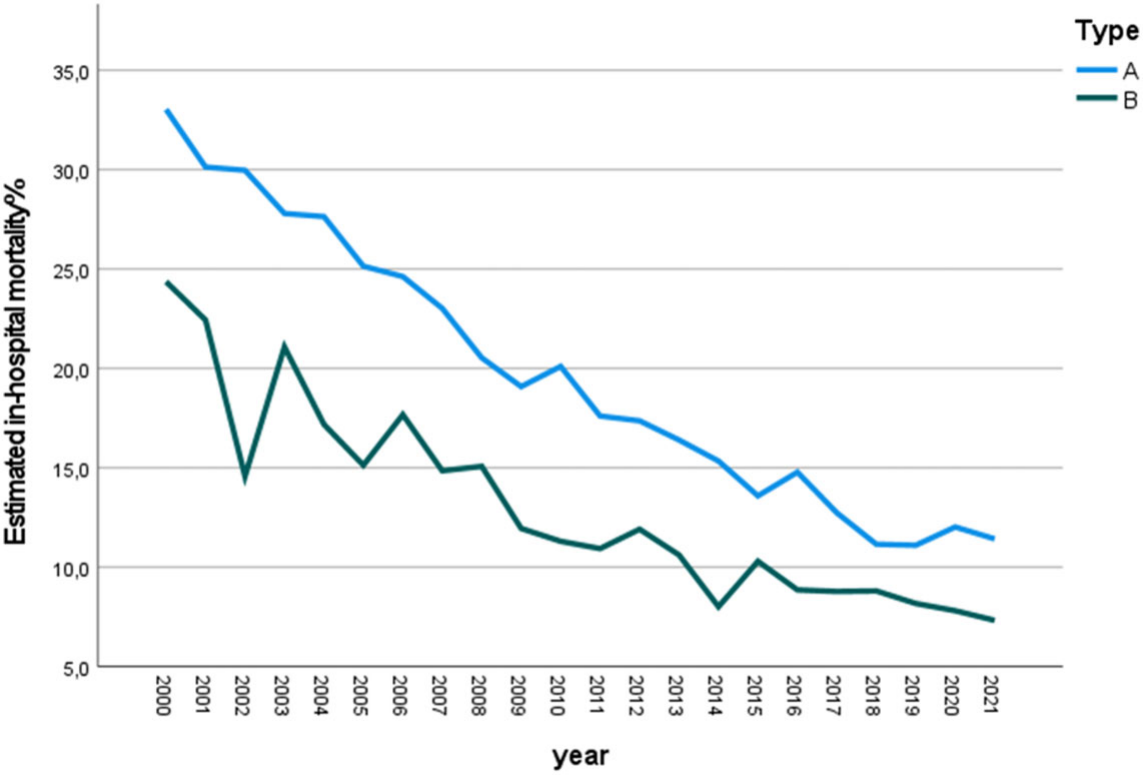
In-hospital mortality trends from 2000 to 2021 in type A acute aortic syndromes and type B acute aortic syndromes.

The estimated in-hospital mortality decreased significantly from 33.06% to 11.45% in TAAAS and from 24.38% to 7.33% in TBAAS from 2000 to 2021 (Fig. 3, *P* < 0.001). However, the trend did not differ between TAAAS and TBAAS (*P* = 0.674).

### Type A AAS

Among patients with TAAAS, 462 (93.5%) were admitted with a diagnosis of aortic dissection, 30 (6.1%) with intramural haematoma and 2 (0.4%) with penetrating aortic ulcer; among patients with TBAAS, 230 (55.0%) had aortic dissection, 100 (23.9%) intramural haematoma and 88 (21.1%) penetrating aortic ulcer.

**Figure 4:**
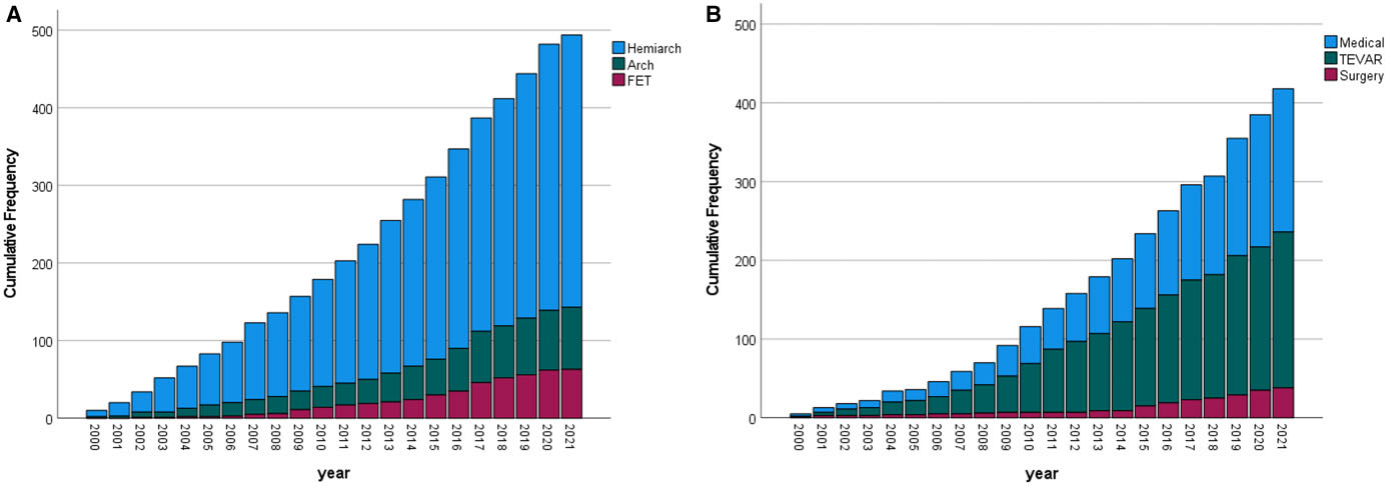
Cumulative frequency of procedures for type A acute aortic syndromes (**A**) and type B acute aortic syndromes (**B**).

All patients with TAAAS who underwent surgery were included in the study: 143 (28.9%) required total arch and 351 (71.1%) hemiarch replacement (Fig. 4A). Notably, in-hospital mortality did not differ between arch and hemiarch repair (22.4% vs 16.5%; *P* = 0.126).

### Type B AAS

Management of TBAAS included medical treatment in 182 (43.5%), endovascular treatment in 198 (47.4%) and open surgery in 38 (9.1%) cases (Fig. 4B).

A remarkably higher mortality was found among aortic dissection (15%) compared with intramural haematoma (4%) and penetrating aortic ulcer (6.8%) (*P* = 0.005). Vice versa, no difference (*P* = 0.170) was detected among patients receiving medical therapy (7.9%), endovascular treatment (11.9%) and surgery (17.1%).

### Long-term outcome

Overall, 404 TAAAS (81.8%) and 376 TBAAS (89.3%) were discharged after the acute phase. The median follow-up was 4.5 years (range 0–19.8) for TAAAS and 2.3 years (range 0–18.6) for TBAAS.

**Figure 5:**
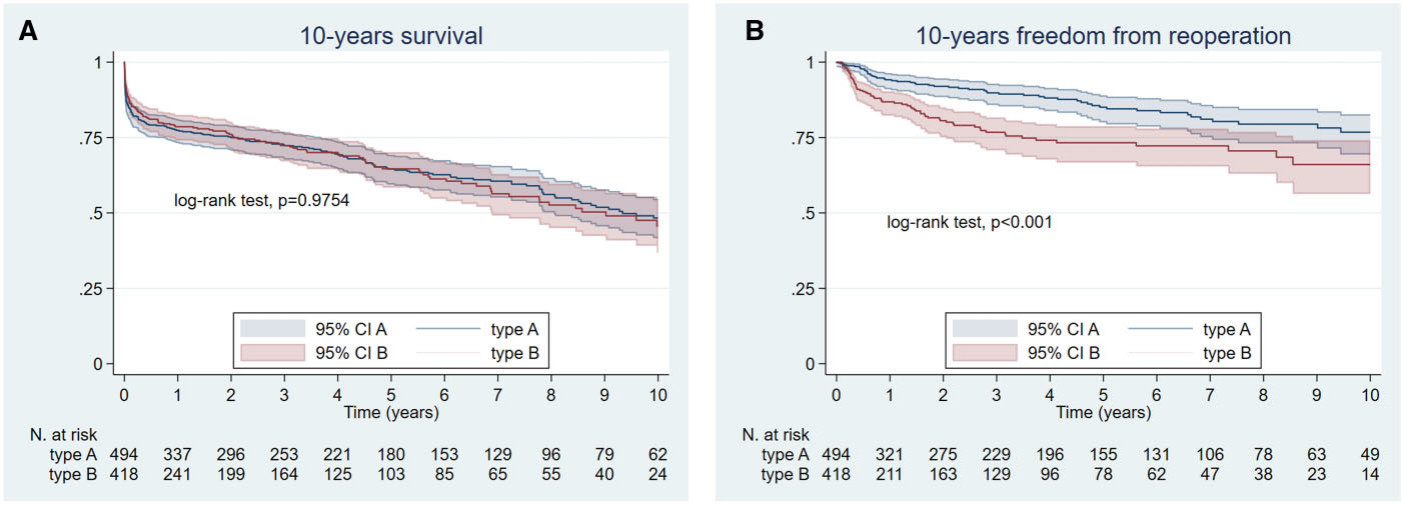
Kaplan–Meier estimates of survival (**A**) and freedom from reoperation (**B**) in type A acute aortic syndromes and type B acute aortic syndromes.

Estimated survival at 1, 5 and 10 years was 74.2%, 62.1% and 45.5% for TAAAS, and 75.4%, 60.7% and 41.0% for TBAAS (log-rank test, *P* = 0.9754) (Fig. 5A).

**Figure 6:**
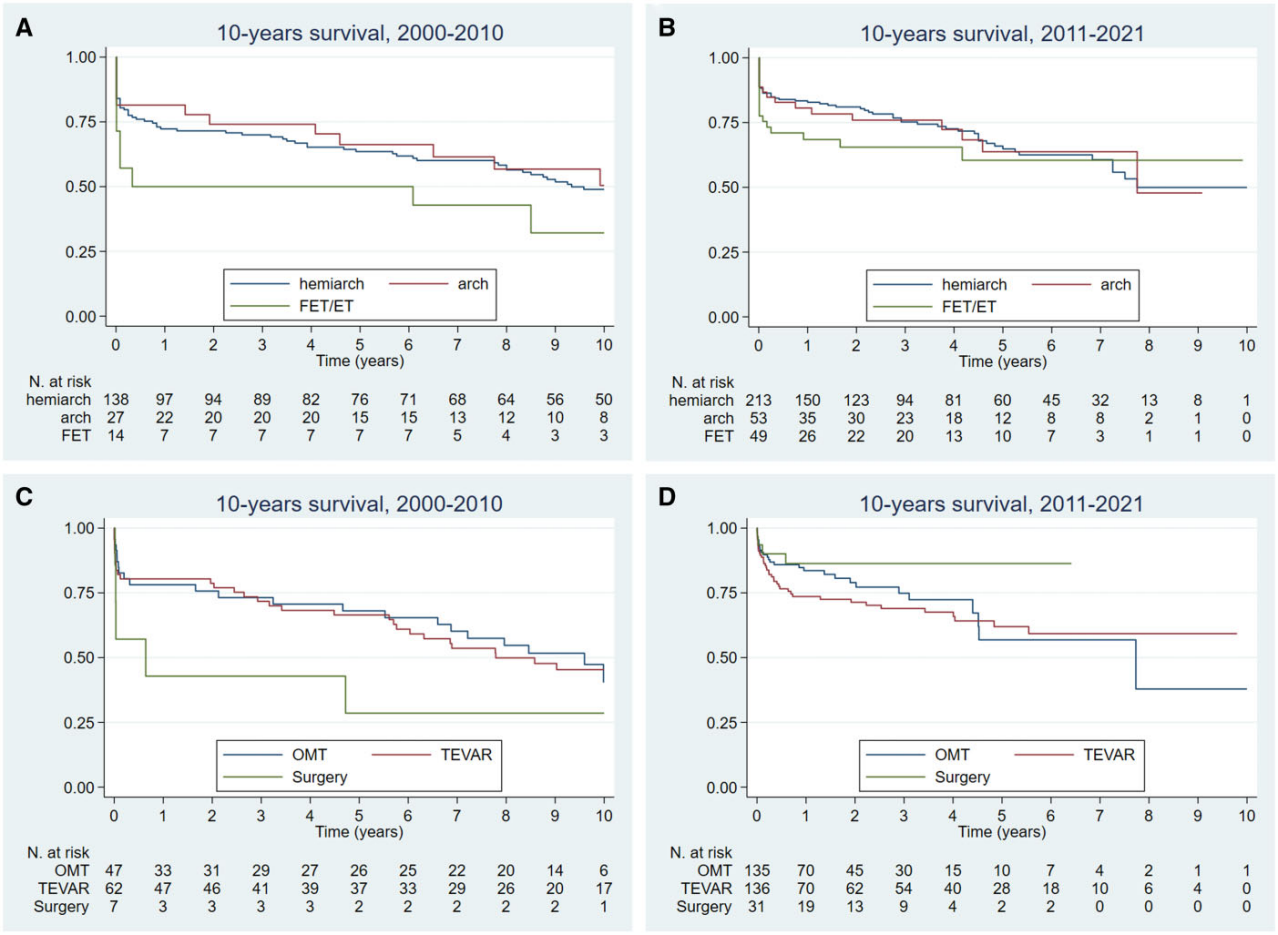
Kaplan–Meier survival estimates for the periods 2000–2010 and 2011–2022 in type A acute aortic syndromes (**A**) and type B acute aortic syndromes (**B**) according to the type of treatment received during the index hospitalization.

Among TAAAS 1-, 5- and 10-year survival was similar between patients who underwent arch repair (80.3%, 64.2% and 49.3%), those who underwent hemiarch repair (78.2%, 65.1% and 48.7%) and those who underwent elephant trunk or frozen elephant trunk (62.7%, 57.5%, 42.0%, log-rank test, *P* = 0.2139) (Fig. 6A and B).

In addition, no difference in survival was found between supracoronary aortic replacement and aortic root replacement (1-, 5- and 10-year survival 75.7%, 63.7%, 47.2% vs 77.9%, 64.4%, 49.2%, log-rank test = 0.06, *P* = 0.811).

As to aetiology, aortic dissection and intramural haematoma showed a similar survival (76.6%, 64.5%, 47.8% vs 78.9%, 53.9% and 53.9%, log-rank test = 0.01, *P* = 0.9416).

Among TBAAS patients treated with medical therapy during index hospitalization, survival at 1, 5 and 10 years (80.9%, 63.7% and 40.5%) did not differ from that of patients undergoing surgical treatment (76.5%, 63.7% and 63.7%), or TEVAR (75.5%, 63,3%, 46.9%; log-rank test = 0.29, *P* = 0.592) (Fig. 6C and D).

Aortic dissection showed a similar 1-, 5- and 10-year survival compared to penetrating aortic ulcer and intramural haematoma (aortic dissection: 76.1%, 64.9%, 49.7%; intramural haematoma: 84.3%, 60.0% and 39.9%; penetrating aortic ulcer 75.9%, 60.5%, 35.6%, log-rank test, *P* = 0.3503).

**Figure 7:**
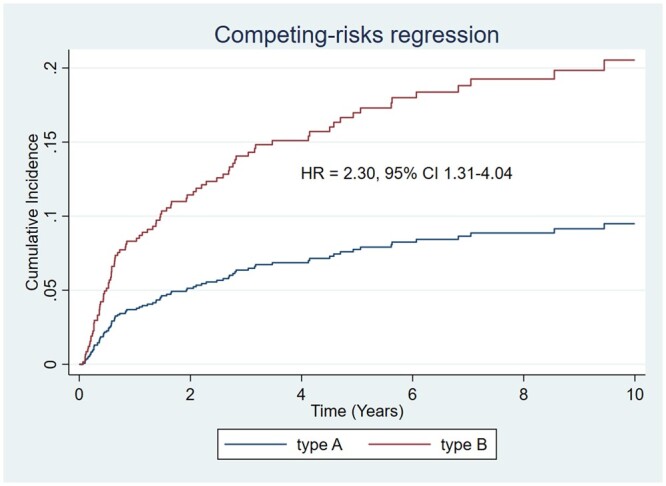
Cumulative risk of reoperation in type A acute aortic syndromes versus type B acute aortic syndromes, adjusted for the propensity score and estimated with competing risk analysis.

Overall, the reoperation rate was significantly lower in TAAAS than in TBAAS (11.7% vs 16.5% *P* = 0.038). Notably, freedom from aortic reoperation in 10 years was significantly higher in TAAAS compared with TBAAS, especially between 1 to 6 years from the baseline intervention (*P* = 0.0001) (Fig. 5B). However, type A patients received much more often later open surgical reinterventions (27/404 vs 3/373) to treat residual dissections in the root and more often in the arch (*n* = 14 frozen elephant trunk procedures). The median surgical follow-up was 2.67 years (interquartile range (IQR) 0.42–6.35) for type A and 1.08 years (IQR 0.11–3.74) for type B. In a competing risk analysis, the cumulative risk of reoperation was 9.5% in TAAAS and 20.5% in TBAAB (hazard ratio (HR) = 2.30, 95% confidence interval (CI) 1.31–4.04) (Fig. 7).

No differences in reoperation rate were found in TAAAS by subtype: 11.9% in aortic dissection vs 13.3% in intramural haematoma (*P* = 0.843), while among TBAAS reoperation rates were similar between aortic dissection and penetrating aortic ulcer (20.4% vs 18.2%) and significantly different from that in intramural haematoma (6%) (*P* = 0.005).

## DISCUSSION

In population-based studies, the annual incidence of acute aortic syndrome ranges between 3.5 and 7.2 cases per 100 000 [7–11]. However, recently a progressively higher incidence of acute aortic syndrome has been reported, due to a surge of elderly patients requiring interventions.

The incidence of acute aortic syndrome in adult US citizens has been investigated in the Rochester Epidemiology Project [10]. Very surprisingly, the study reported that since 1995 the incidence of acute aortic syndrome was stable over time. However, they found an increase in population age and incidence of penetrating aortic ulcer (from 0.6 to 2.6 per 100 000 person-year) compared to intramural haematoma and aortic dissections [10]. In a similar Italian population-based study the annual incidence rate was 4.7 per 100 000 inhabitants [11]. This value remained stable over time, although it confirmed a growing population age, as 20% of the dissected patients were 80 years or older.

An overview of acute aortic dissections in the last 22 years is provided by the International Registry for Aortic Dissection, including >50 aortic centres [12]. Elderly population was stratified by age and therapeutic strategy, and 30% of the population was older than 70 years. Older patients were more likely to receive medical therapy than surgical procedures, however, the proportion of patients in their 70s and 80s who underwent surgery tended to increase over time because endovascular and hybrid therapies were also considered. Overall in-hospital mortality was higher in the octogenarians, but surgical mortality was similar (25.1% vs 21.7%) among groups, as well as post-operative complications including stroke, probably because of a better patient selection and less invasive operative strategies [12].

In our population, characteristics of patients with TAAAS and TBAAS were different, with TBAAS patients being significantly older (68 vs 65 years) and with more comorbidities. As to the volumes of patients, regardless the type of acute aortic syndrome, a steady increase of referrals was observed over time, probably due to centralization of patient management.

Overall in-hospital mortality was 14.8% and it substantially decreased over time (Fig. 3) as a result of the change in management and surgical approach to these patients. Indeed, outcomes in type A dissection significantly improved as regards myocardial infarction, kidney injury and coma. The explanation can be found in the possibility to have hybrid surgical suites to rapidly address organ malperfusion, along with a wider use of the frozen elephant trunk and more experience with different arterial cannulation sites (including the carotid aa) for CPB. Among the different types of acute aortic syndrome, aortic dissection remained the most prominent condition, with a higher in-hospital mortality compared to intramural haematoma and penetrating aortic ulcer. This was especially true for type A compared to type B aortic dissection.

Over time, TAAAS received more often a hemiarch procedure without differences in survival. Conversely, TBAAS was more often managed with endovascular treatment, followed by medical therapy alone and surgical repair in selected and older patients [13]. In both types of acute aortic syndrome in-hospital mortality was similar despite different treatment options. However, during the last decade in our Institution the frozen elephant trunk procedure has been more frequently used as first-line treatment when the primary entry tear was located in the arch and in case of distal malperfusion.

Our findings suggest that 2 major factors contributed to the improvement of early outcomes: one is the improvement in endovascular techniques. Stent grafts are often used as a definitive treatment in TBAAS or to address complications of TAAAS. Moreover, it is now possible to be more aggressive in the arch with previous rerouting of 1 or more epiaortic vessels or using bail-out procedures with fenestrated or branched endografts. The progressive use of endoprostheses has been also described in TAAAS open arch surgery, especially with the introduction of antegrade stent graft deployment in the descending thoracic aorta [14]. Different authors reported many options to facilitate rerouting of epiaortic vessels to shorten operative times, including self-made hole into the graft [15], covered stent inside the left subclavian artery [16] and Supra-Aortic Vessel anastomosis STEnt Bridging (SAVSTEB) technique to move even more proximally the distal anastomosis in the arch [17].

The second important factor that improved the outcomes of acute aortic syndrome is a better understanding of dissections and centralization of care. In order to facilitate triage and improve treatment outcomes, 2 new classifications have now been proposed. The Type Entry Malperfusion (TEM) classification is based on the type of dissection (introducing the concept of ‘non-A non-B’ acute aortic syndrome), the location of the primary entry tear and the malperfusion status [18]. On the other hand, the STS/AATS classification [6] considers type A any aortic dissection with an entry tear in zone 0, while type B includes any aortic dissection with an entry tear in zone 1 or a more distal aortic zone. It also includes a subclassification to exactly describe proximal and distal extension to facilitate any endovascular treatment. It allows a better understanding of the pathology and facilitates treatment.

Specifically, in this study, 47.4% of TBAAS were treated with endovascular repair and 9.1% with surgical repair. Our approach during time progressively changed in favour of pre-emptive TEVAR and the use of the frozen elephant trunk procedures in selected subgroups of patients at high risk for a proximal type I endoleak or retrograde acute aortic dissection.

During follow-up, survival was similar between TAAAS and TBAAS. The 10-year Kaplan–Meier estimate confirmed the high mortality of these conditions with <50% of patients surviving in the long-term. Concerning the type of treatment, a more aggressive aortic arch replacement in TAAAS was not superior to hemiarch replacement and an initial medical therapy was not inferior to endovascular treatment in TBAAS.

On the other hand, TBAAS has a much higher rate of reinterventions compared to TAAAS with a cumulative risk of 9.5% in TAAAS and 20.5% in TBAAS (HR = 2.30, 95% CI 1.31–4.04). This is probably the result of the insufficient first endorepair, intrinsic to the procedure itself (repair rather than replace), that often necessitates of later multiple re-repair for endoleaks or distal aortic disease.

Undoubtedly, pre-operative imaging (investigating number and location of entries, aortic diameters, patency of false lumen, etc.) plays a crucial role in the initial management of these patients [19]. The possibility to predict distal aortic remodelling should be considered to reduce later reinterventions. In a Korean study [20], pre-operative CT parameters at the level of the distal anastomosis were compared between patients with late positive remodelling of the proximal portion of the descending thoracic aorta and those without remodelling. Results showed that a positive distal aortic remodelling was strongly correlated with small false lumen at the distal anastomotic zone and with the lack of residual arch branches with a patent false lumen [20]. Implications are that some elderly patients can be spared from the aggressive total arch replacement and still have favourable late outcomes.

### Limitations

Our study has several limitations, the first being its retrospective nature. However, patients were prospectively included in the dataset of a single centre with a careful clinical and radiologic data collection. The second limitation is that the impact of changes in surgical indications and related therapeutic options over time could not be assessed. The third limitation is that data on non-surgical candidates are not recorded in the registry. Therefore, outcomes of these patients are not available.

## CONCLUSION

Better patient triage and surgical/endovascular techniques led to substantial improvements in the management and outcomes of acute aortic syndromes, since the introduction of this nosological entity in the early 2000, with reduction in early mortality and reoperation rate.

However, acute aortic syndrome remains a life-threatening medical condition, with a long-term mortality of over 50%, where endovascular and surgical techniques will continue to be used alternatively or in combination.

## Supplementary Material

ezad350_Supplementary_Data

## Data Availability

The data underlying this article will be shared on reasonable request to the corresponding author. **Giacomo Murana:** Conceptualization; Investigation; Validation; Writing—original draft; Writing—review & editing. **Gregorio Gliozzi:** Data curation; Investigation; Writing—original draft; Writing—review & editing. **Paola Rucci:** Formal analysis; Methodology; Supervision; Validation; Writing—review & editing. **Daniela Votano:** Data curation; Writing—original draft. **Valentina Orioli:** Data curation; Writing—original draft. **Simona Rosa:** Formal analysis; Writing—review & editing. **Gianluca Folesani:** Conceptualization; Investigation; Writing—review & editing. **Francesco Buia:** Data curation; Investigation; Writing—review & editing. **Luigi Lovato:** Investigation; Validation; Writing—review & editing. **Davide Pacini:** Conceptualization; Supervision; Validation; Writing—review & editing.

## Notes

Presented at the AATS—Aortic Symposium, Boston, MA, USA, 13–14 May 2022.

## ABBREVIATIONS

CT: Computed tomography
TAAAS: Type A acute aortic syndromes
TBAAS: Type B acute aortic syndromes
