# Supplementary material for: Survival and reoperation in acute aortic syndromes—a single-centre experience of 912 patients
Source: Eur J Cardiothorac Surg. 2023 Oct 25;64(5):ezad350. doi: 10.1093/ejcts/ezad350 (PMC10722879; doi:10.1093/ejcts/ezad350)
Supplement: ezad350_Supplementary_Data [file ezad350_supplementary_data.docx]

**Supplementary material**

Supplementary Table 1 shows that patients with TBAAS were significantly older (mean age of 68.4+12.7 years vs. 65.4+11.7 in TAAAS, p<0.001). Patients with intramural hematoma and penetrating aortic ulcer were older than those with aortic dissection both in TAAAS and in TBAAS.

In addition, patients with TBAAS had more cardiovascular risk factors (hypertension 75.1% vs. 82.5%, p=0.008; smoking history 29.1% vs. 42.8%, p<0.001; diabetes mellitus 4.5% vs. 8.9%, p=0.01), more comorbidities (coronary artery disease 6.7% vs. 10.5%, p=0.042; chronic kidney disease 6.5% vs. 13.6%, p<0.001) and previous cardiac surgery (2.8% vs. 8.6%, p<0.001); pre-operative cerebrovascular accident (transient ischemic attack/stroke) were more frequent in TAAAS (18.6 % vs. 0.7%, p<0.001).

**Supplementary Table 1. Patient characteristics in TAAAS and TBAAS**

| **Baseline features** | **TAAAS**  **(n=494)** | **TBAAS**  **(n=418)** | *p-value* |
| --- | --- | --- | --- |
| Age, years *(mean, sd)* | 65.4 ± 11.7 | 68.4 ± 12.7 | **<0.001** |
| *Age by type** |  |  |  |
| AD *(mean, sd, n)* | 65.0 ± 11.9 462 | 64.5 ± 13.6 230 |  |
| IMH *(mean, sd, n)* | 71.3 ± 7.4 30 | 73.5 ± 10.3 100 |  |
| PAU *(mean, sd, n)* | 74.3 ± 4.0 2 | 72.8 ± 8.6 88 |  |
| BMI, kg/m^2^ *(mean, sd)* | 26.6 ± 4.4 | 26.3 ± 4.4 | 0.299 |
| LVEF *(mean, sd, n)* | 58.1 ± 6.3 | 60.3 ± 7.0 | **<0.001** |
| Gender, male *(n, %)* | 331 (67.0) | 300 (71.8) | 0.131 |
| Hypertension *(n, %)* | 371 (75.1) | 345 (82.5) | **0.008** |
| Smoking history *(n, %)* | 144 (29.1) | 179 (42.8) | **<0.001** |
| Diabetes mellitus *(n, %)* | 22 (4.5) | 37 (8.9) | **0.010** |
| CAD *(n, %)* | 33 (6.7) | 44 (10.5) | **0.042** |
| TIA/Stroke *(n, %)* | 92 (18.6) | 3 (0.7) | **<0.001** |
| Previous cardiac surgery *(n, %)* | 14 (2.8) | 37 (8.9) | **<0.001** |
| CKD *(n, %)* | 32 (6.5) | 57 (13.6) | **<0.001** |
| COPD *(n, %)* | 43 (8.7) | 40 (9.6) | 0.729 |
| AR ≥ moderate | 148 (30.0) | 17 (4.1) | **<0.001** |
| Dyslipidemia | 77 (15.6) | 137 (32.8) | **<0.001** |
| Marfan syndrome | 4 (0.8) | 9 (2.2) | 0.100 |
| Syncope at presentation | 47 (9.5) | 25 (6.0) | **0.050** |
| Coma at presentation | 11 (2.2) | 0 (-) | **0.001** |
| Malperfusion | 123 (24.9) | 129 (30.9) | 0.053 |
| **Follow up** |  |  |  |
| PND | 67 (13.6) | 1 (0.2) | **<0.001** |
| MI | 12 (2.4) | 7 (1.7) | 0.491 |
| AKI | 146 (29.6) | 24 (5.7) | **<0.001** |
| Dialysis | 124 (25.1) | 16 (3.8) | **<0.001** |
| SCI | 3 (0.6) | 8 (1.9) | 0.124 |
| Coma | 32 (6.5) | 0 (-) | **<0.001** |

*Age in type A syndromes: ANOVA F=4.77, p=0.009, significant post-hoc comparisons, IMH vs. AD, p=0.012; age in type B syndromes: ANOVA F: 28.5, p<0.001; significant post-hoc comparisons: IMH and PAU vs. AD, p<0.001).

|  |
| --- |

AD: aortic dissection; AKI: acute kidney injury; AR: aortic regurgitation; BMI: body mass index; CAD: coronary artery disease; CKD: chronic kidney disease; COPD: chronic obstructive pulmonary disease; IMH: intramural hematoma; LVEF: left ventricle ejection fraction; MI: myocardial infarction; PAU: penetrating aortic ulcer; PND: permanent neurological deficit; SCI: spinal cord ischemia; TIA: transient ischemic attack.
